# Supplementary material for: Click-free imaging of carbohydrate trafficking in live cells using an azido photothermal probe
Source: Sci Adv. 2024 Aug 21;10(34):eadq0294. doi: 10.1126/sciadv.adq0294 (PMC11338237; doi:10.1126/sciadv.adq0294)
Supplement: Supplementary file 1 — Supplementary Texts 1 to 4 Figs. S1 to S25 Table S1 References [file sciadv.adq0294_sm.pdf]

Science Advances
AAAS

Supplementary Materials for

Click-free imaging of carbohydrate trafficking in live cells using an azido photothermal probe

Qing Xia *et al.*

Corresponding author: Ji-Xin Cheng, jxcheng@bu.edu; Mingdi Yan, mingdi_yan@uml.edu

Sci. Adv. **10**, eadq0294 (2024)
DOI: 10.1126/sciadv.adq0294

This PDF file includes:

Supplementary Texts 1 to 4
Figs. S1 to S25
Table S1
References

Supplementary Text

Supplementary Text 1. Synthetic procedures of TreAz.

The synthetic procedure of 6-azido-trehalose (TreAz) is shown in **Fig. S21**. The detailed characterizations of product and intermediates are reported in previous work (*24*).

Supplementary Text 2. MIP spectra acquisition and processing workflow.

The MIP spectra for both solutions and bacteria were obtained from the pinpoint MIP spectra using previous method (*62*). To acquire pinpoint MIP spectra of solutions, the focused visible beam was centered in the solution based on the transmitted image, and the mid-IR laser continuously swept wavenumbers from 2,000 cm^{-1} to 2,300 cm^{-1} at a speed of 50 cm^{-1}/s. The raw MIP spectra were obtained from a lock-in amplifier with a time constant of 20 ms. Since both DMSO and water have the absorbance in this region, all MIP spectra in the cell-silent window underwent a subtraction of solvent background or background from the control samples. The spectra then further underwent the baseline correction and smoothing with the Savitzky-Golay method using Origin. The final MIP spectra were normalized by the IR power profile in the corresponding region. For pinpoint MIP spectra of bacteria, both the cell surface and cell cytosol were selected as target points of interest based on the transmitted images, and control spectra were acquired from untreated bacteria. The workflow is shown in **Fig. S22**.

To investigate the biological systems with large sizes, it is hard to select the area for acquiring pinpoint MIP spectra of App treated samples. Thus, all the MIP spectra for yeast and HeLa cells were obtained from the hyperspectral MIP images. The hyperspectral scanning range is from 2,060 cm^{-1} to 2,220 cm^{-1} with a step size of 2 cm^{-1}/frame. The intracellular MIP spectra were then obtained from the selected region of interest (typically 25 pixels) in the hyperspectral MIP imaging stack. The final MIP spectra were obtained by background subtraction, baseline correction, smoothing and normalized by the IR power profile in the corresponding region. The workflow is shown in **Fig. S23**.

Supplementary Text 3. MIP image processing method.

To obtain the MIP images of App treated bacteria at cell silent window, water background subtraction was performed using previous method (*62*). To remove the water background, two selected frames at the azide peak of TreAz at 2,180 cm^{-1} and the water infrared absorbance at 2,100 cm^{-1} were collected, respectively. The wavenumbers of 2,100 cm^{-1} and 2,180 cm^{-1} were selected based on the MIP spectra of TreAz treated bacteria and control bacteria from both of the cell surface and cytosol (**Fig. S6**). The azide treated bacteria show an obvious peak at 2,180 cm^{-1} above the background absorption. The MIP signal intensity in the control sample at 2,100 cm^{-1} is equal to the MIP signal intensity at azide peak of 2,180 cm^{-1} in the same control sample. After subtraction, the bacteria in the control groups exhibit no signal (**Fig. 2G**). Thus, all MIP images of bacteria were collected at 2,180 cm^{-1} and underwent a subtraction process using MIP images collected at 2100 cm^{-1} to remove the water background and to generate the final MIP images. To enhance signal-to-noise ratio, the images at all channels were obtained via the averaging of 10 frames.

For MIP imaging of yeast and HeLa cells, the hyperspectral MIP images were denoised using a BM4D V3.2 (http://www.cs.tut.fi/~foi/GCF-BM3D/index.html#ref_software) denoising algorithm (*63, 64*) via MATLAB. Then, water background subtraction was applied to the single-color MIP image based on the corresponding MIP spectra of the yeast cells (**Fig. S14**). For 6-Gal

treated and control yeast, MIP images in the azide channel were generated by subtracting the MIP image at 2,156 cm^{-1} (azide peak) from the half sum of the MIP images at 2,130 cm^{-1} and 2,170 cm^{-1} (water absorbance). For 1-Gal treated yeast, MIP images in the azide channel were generated by subtracting the MIP image at 2,130 cm^{-1} (azide peak) from the half sum of the MIP images at 2,110 cm^{-1} and 2,150 cm^{-1} (water absorbance). For azide treated and control HeLa cells, MIP images in the azide channel were generated by subtracting the MIP image at 2,140 cm^{-1} (azide peak) from the half sum of the MIP images at 2,120 cm^{-1} and 2,160 cm^{-1} (water absorbance) according to the MIP spectra of the cells (**Fig. S16**).

Supplementary Text 4. Click-reaction-based fluorescence imaging.

The clickable fluorescent dye used in this work is a commercial product **AF 488 DBCO**. The alkyne group in dibenzocyclooctyne (DBCO) can react with the azide group in azide-tagged molecules, such as TreAz, and form a stable triazole via strain promoted alkyne-azide cycloaddition (*65*). The scheme is illustrated in **Fig. S24**.

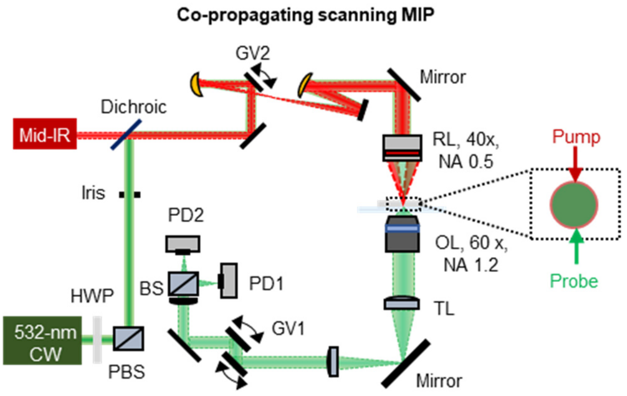

Fig. S1. Schematic illustration of the co-propagating scanning MIP imaging system. The visible probe was co-aligned with the pump mid-IR laser and focused by a reflective objective (RL). Probe photons were collected in forward direction. The intensity was sensed by a silicon photodiode (PD). PD1 was used to acquire the transmission and PD2 was used to acquire the MIP image. BS: T90:R10 beam splitter, GV1 and GV2: galvo mirrors, HWP: half-wave plate, NA: numerical aperture, OL, objective lens, PBS: polarized beam splitter, TL: tube lens. Inside the dotted box is the schematic illustration of the beam size of pump and probe.

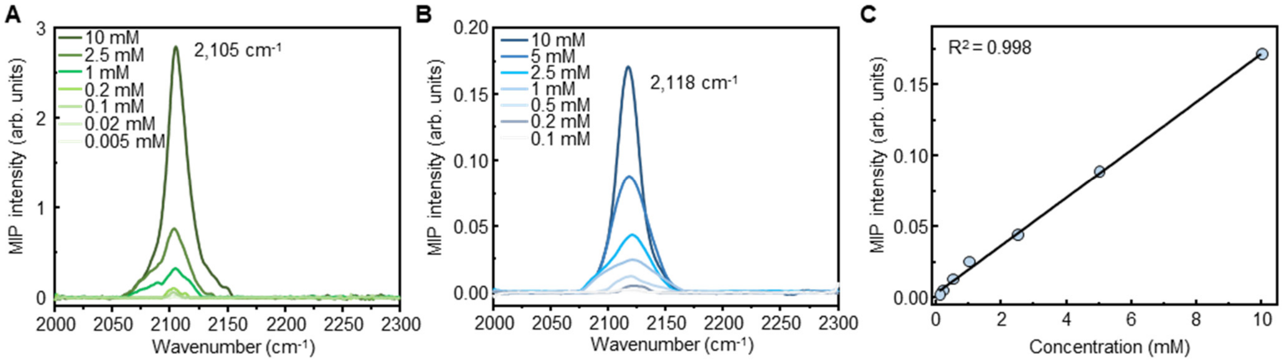

Fig. S2. Limit of detection (LOD) of TreAz. (**A**) MIP spectra of TreAz at different concentrations in DMSO. (**B**) MIP spectra of TreAz at different concentrations in PBS. (**C**) MIP signal intensity of TreAz at different concentrations in PBS.

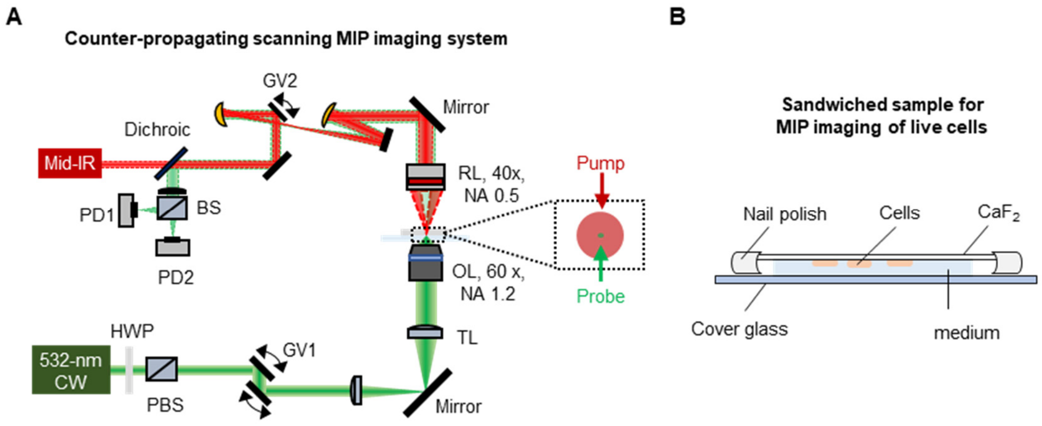

Fig. S3. Schematic illustration of counter-propagating scanning MIP imaging system. (**A**) MIP microscope setup. The IR pump beam was generated by a tunable (from 900 to 2,300 cm^{-1}) quantum cascade laser. The visible probe was provided with a continuous-wave 532 nm laser. Probe photons were collected in forward direction. The intensity was sensed by a silicon photodiode (PD). PD1 was used to acquire the transmission and PD2 was used to acquire the MIP image. BS: T90:R10 beam splitter, GV1 and GV2: galvo mirrors, HWP: half-wave plate, NA: numerical aperture, OL, objective lens, PBS: polarized beam splitter, RL: reflective objective, TL: tube lens. Inside the dotted box is the schematic illustration of the beam size of pump and probe. (**B**) Illustration of sandwiched sample for MIP imaging of live cells. Top substrate is CaF_2, and the bottom substrate is No. 1 glass cover slide. Cells were sandwiched between two substrates and immersed in medium.

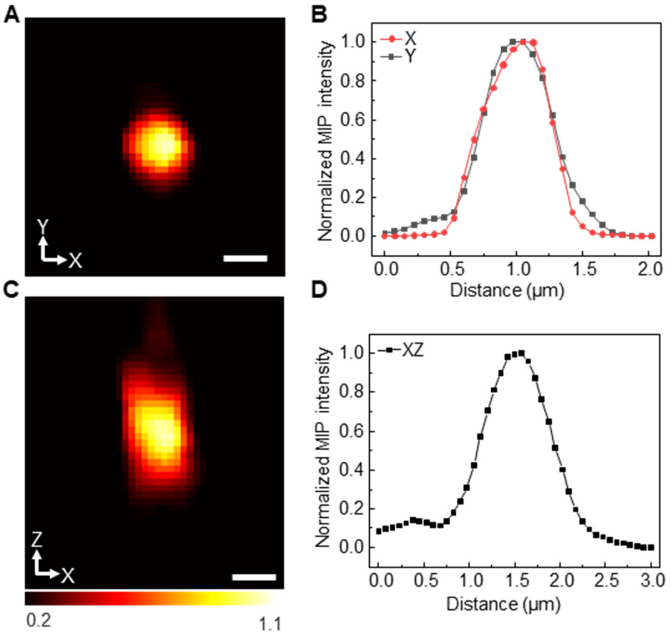

Fig. S4. Spatial resolution of the counter-propagating MIP imaging System. (**A**) MIP image of a 500-nm diameter PMMA particle acquired with IR excitation at 1,729 cm^{-1}. (**B**) Line profiles of MIP intensity along X and Y axes, with fitted full width at half maximum (FWHM) of 578 nm (X) and 584 nm (Y), respectively. (**C**) Projection of the same particle along the XZ axis. (**D**) Line profiles of MIP intensity along XZ axis, with a fitted FWHM of 823 nm. The 3D point spread function is deconvolved using the image with the actual particle size along the three principal axes, resulting in a lateral resolution of 290 nm (X) and 302 nm (Y), and an axial resolution of 653 nm. Scale bars: 500 nm.

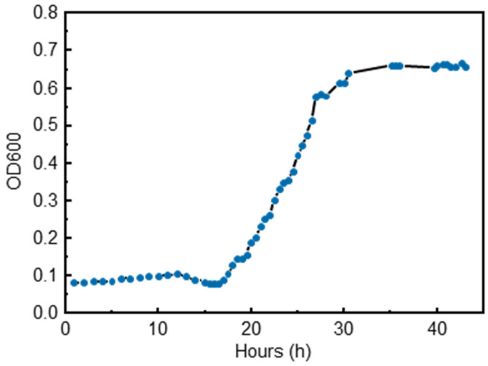

Fig. S5. A representative growth curve of *M. smeg*. OD600 represents optical densities measured at 600 nm wavelength.

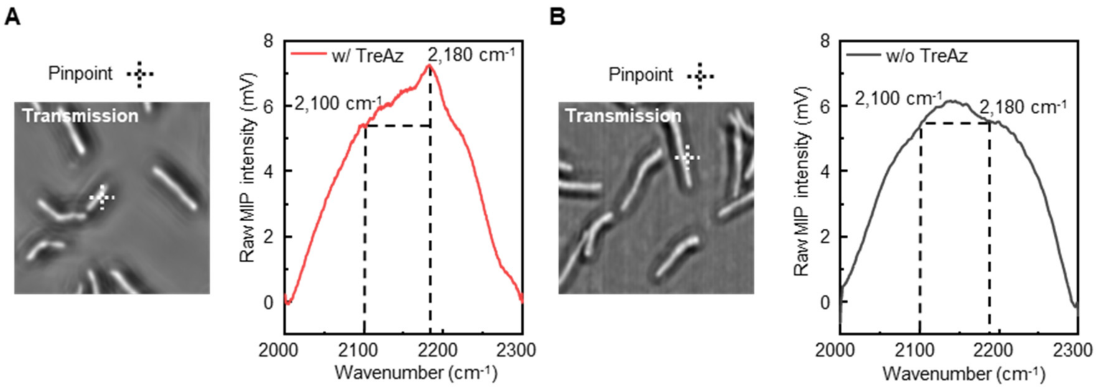

Fig. S6. Raw pinpoint MIP spectra of *M. smeg* in the silent region. MIP spectra acquired from (**A**) TreAz treated *M. smeg* and (**B**) control *M. smeg*. The spectra shown here are raw data without water background subtraction.

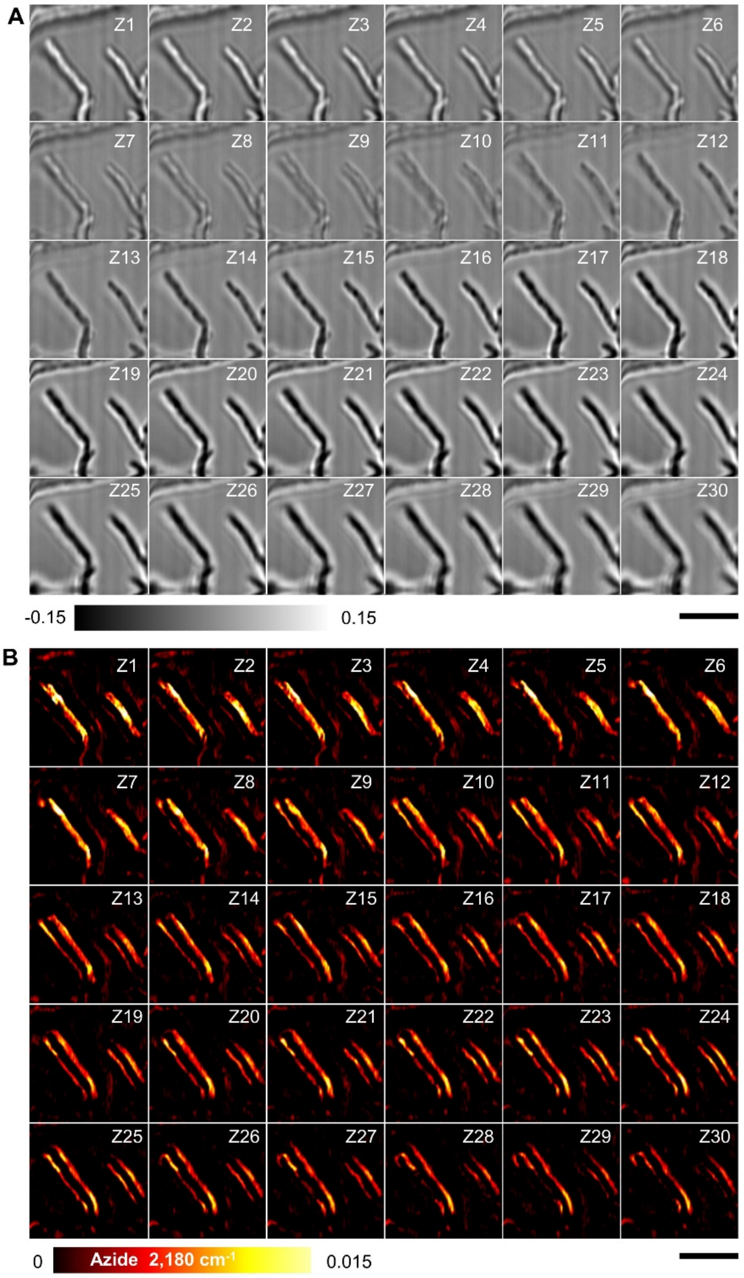

Fig. S7. MIP Z stack imaging demonstrates TreAz localization to the membrane. (**A**) Multi-stack transmission images of TreAz treated *M. smeg*. (**B**) Multi-stack MIP imaging of TreAz treated *M. smeg* at the azide channel. The Z1 image indicates the bottom of the bacteria. The Z step size is 100 nm. Scale bar: 10 µm.

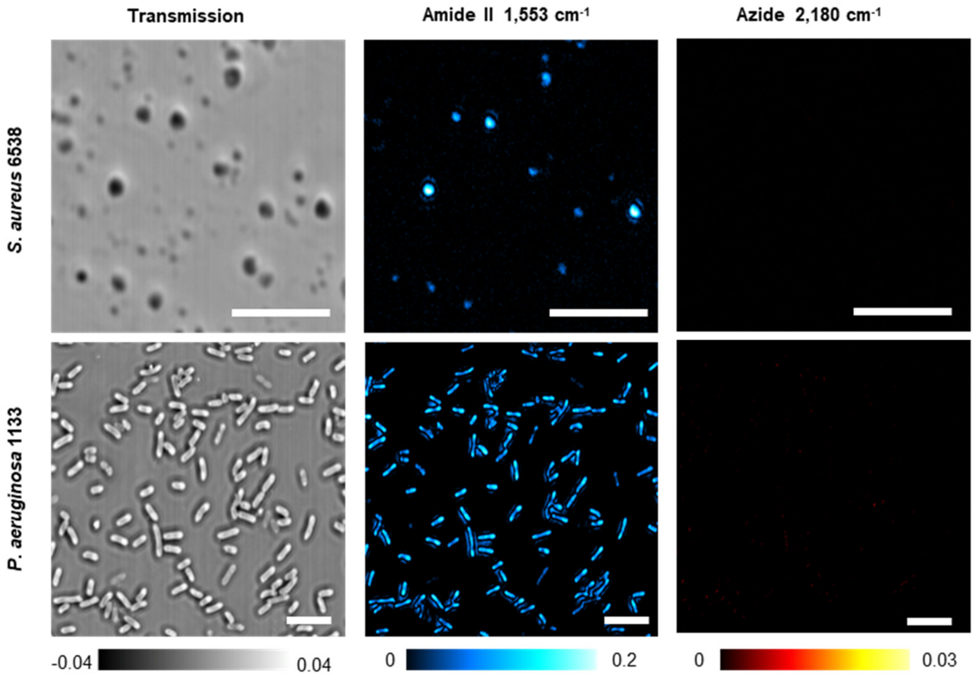

Fig. S8. MIP imaging of control bacterial strains treated with TreAz. Gram-positive *S. aureus* 6538 and Gram-negative *P. aeruginosa* 1133 were both treated with 50 µM TreAz for 1 hour prior to MIP imaging. Scale bars: 10 µm.

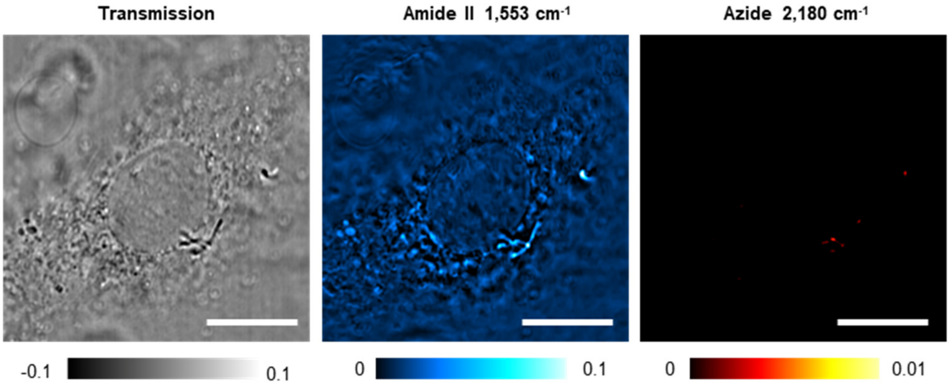

Fig. S9. MIP imaging of untreated *M. smeg* deposited on lung A549 cells. Scale bars: 20 µm.

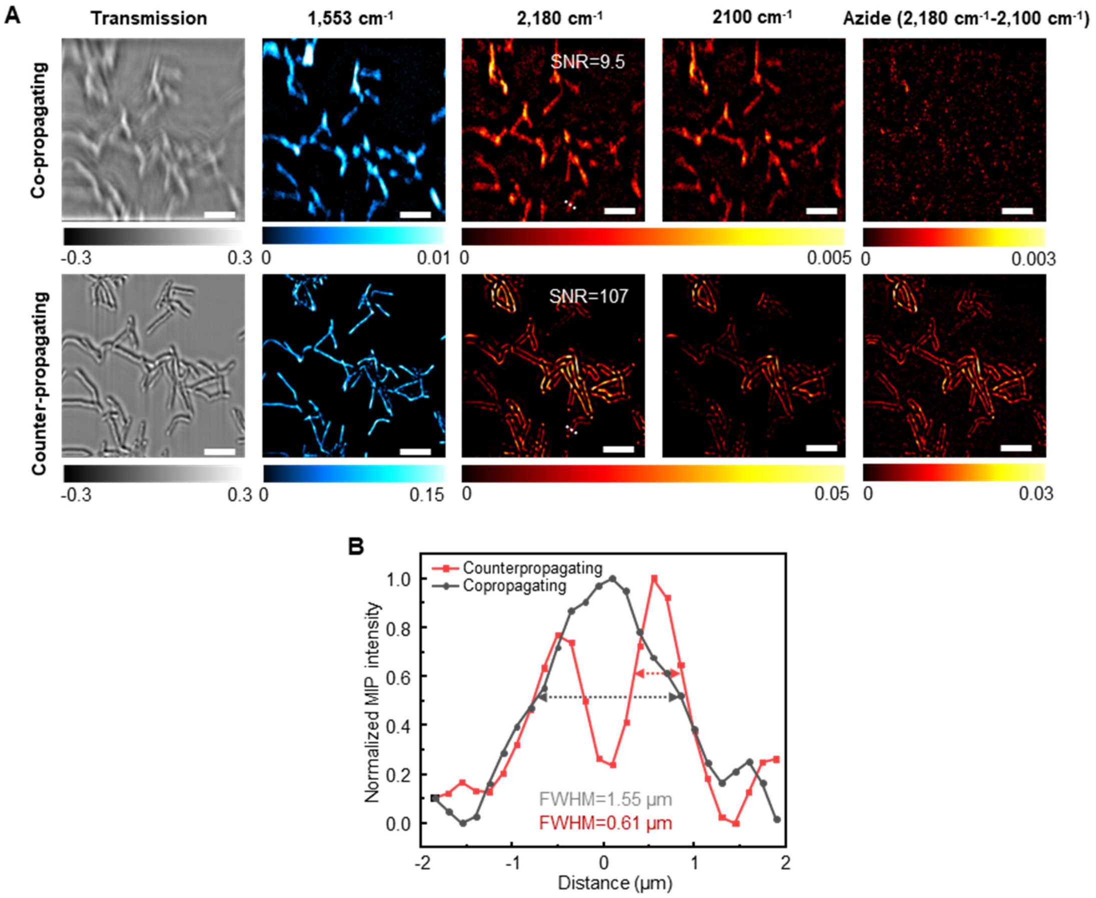

Fig. S10. Head-to-head comparison between counter-propagating and co-propagating MIP imaging of TreAz treated *M. smeg*. (**A**) Multicolor MIP imaging of TreAz treated *M. smeg*. Treatment time: 26 h. The signal-to-noise ratio (SNR) level at 2180 cm^{-1} was calculated from the ratio between the mean value of selected cell region (4 pixels) and the standard deviation of the selected background region (4 pixels) in single color MIP imaging at 2,180 cm^{-1}. Scale bars: 5 µm. The MIP images at 1553 cm^{-1} shows protein distribution. After subtracting the off-resonance image at 2110 cm^{-1} from the on-resonance image at 2180 cm^{-1}, the azide signal from the mycobacterial membranes is buried in the noise in the co-propagating system but is clearly seen in the counter-propagating system, showing the significance of using a tightly focused probe beam to sense nanoscale objects in MIP microscopy. (**B**) Line profiles of MIP intensity along the cell body labeled with dotted white lines in panel a (2180 cm^{-1}). The full width at half maximum (FWHM) is 1.55 µm in co-propagating imaging and 0.61 µm in counter-propagating, respectively.

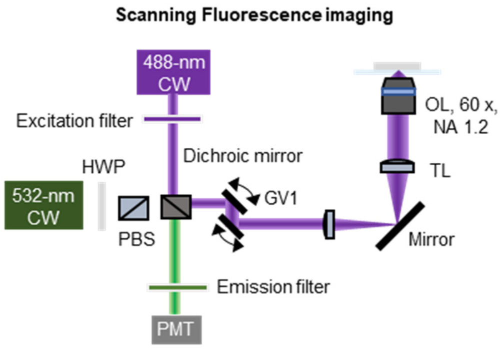

Fig. S11. The lab-built fluorescence imaging modality on the MIP microscope. The excitation light was provided with a continuous-wave 488 nm laser. Emission was collected in epi-detection via a photomultiplier tube (PMT). The transmitted image was collected with the illumination of 532 nm laser via the same pathway for MIP detection in **Fig. S3**. GV1: galvo mirrors, HWP: half-wave plate, NA: numerical aperture, OL: objective lens, PBS: polarized beam splitter, TL: tube lens.

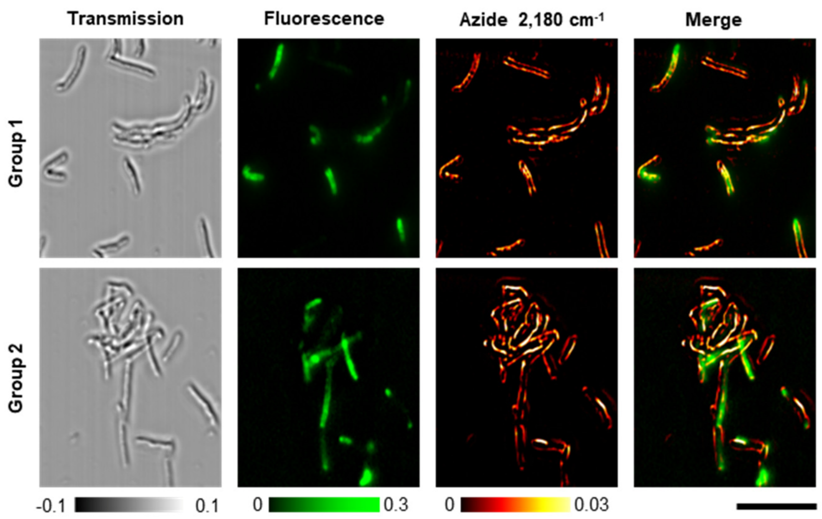

Fig. S12. Two independent repeats of colocalized MIP and fluorescence imaging of single *M. smeg*. The bacteria were treated with 50 μM TreAz till the logarithmic phase, then underwent click reaction following a standard protocol of click chemistry. Scale bar: 20 μm.

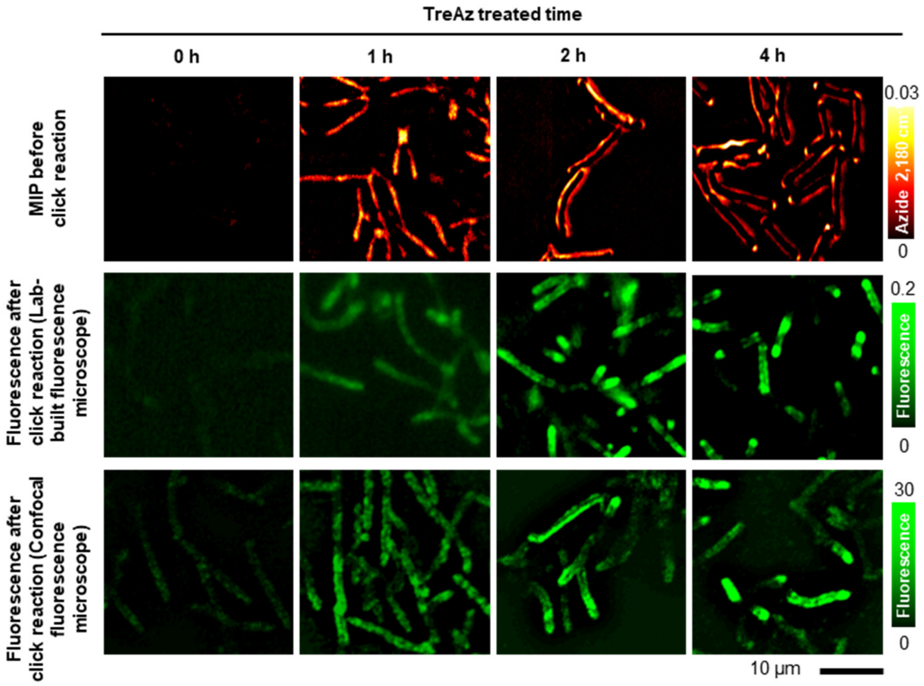

Fig. S13. MIP and fluorescence imaging of TreAz treated *M. smeg* with different incubation times. Fluorescent mages were acquired on a lab-built fluorescence microscope and a commercial confocal fluorescence microscope, respectively. Scale bar, 10 µm.

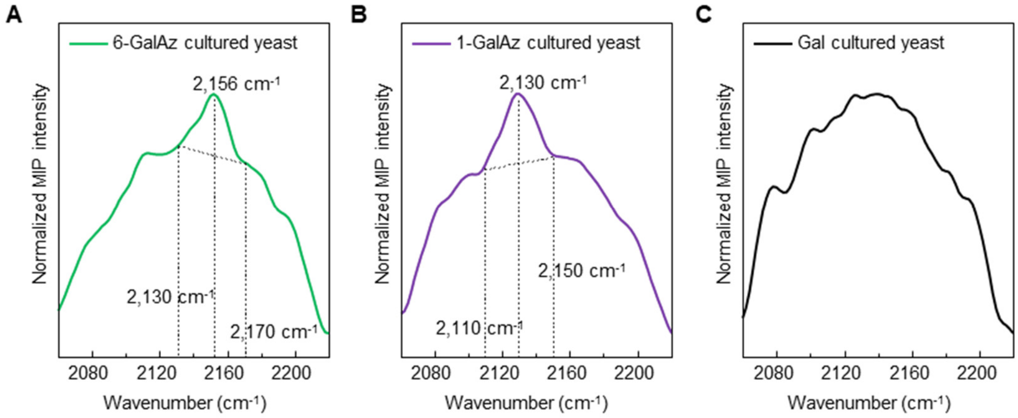

Fig. S14. Raw MIP spectra of yeast cells in the silent region. MIP spectra acquired from (**A**) 6-GalAz, (**B**) 1-GalAz, and (**C**) Gal cultured yeast cells. The spectra are raw data without water background subtraction.

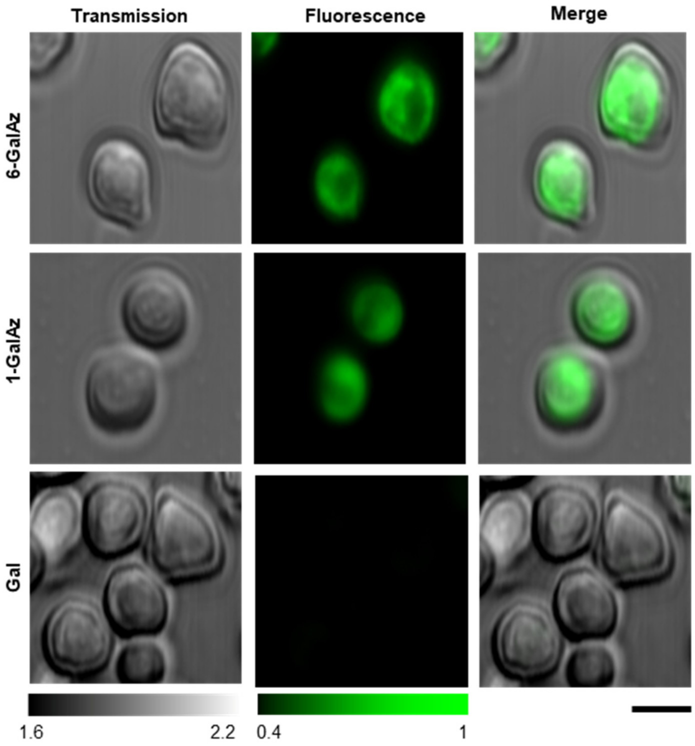

Fig. S15. Fluorescence images of single yeast cells cultured with 6-GalAz, 1-GalAz, Gal and underwent click reactions. Scale bar: 5 μm.

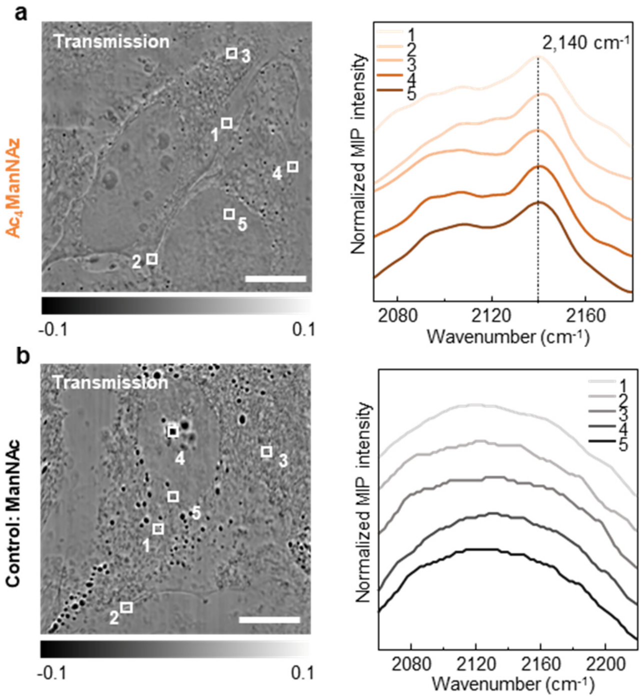

Fig. S16. Raw MIP spectra at indicated positions of HeLa cells in the silent region. MIP spectra acquired from (**A**) $Ac_4ManNAz$ and (**B**) ManNAc treated HeLa cells shown in **Fig. 6D**. Scale bars: 20 μm. White boxes labeled in the transmission indicate the selected region (25 pixels) where the spectra were obtained. The spectra shown here are raw data without water background subtraction. Box 1, 2 in (**A**) and box 2 in (**B**) indicated the position of membranes.

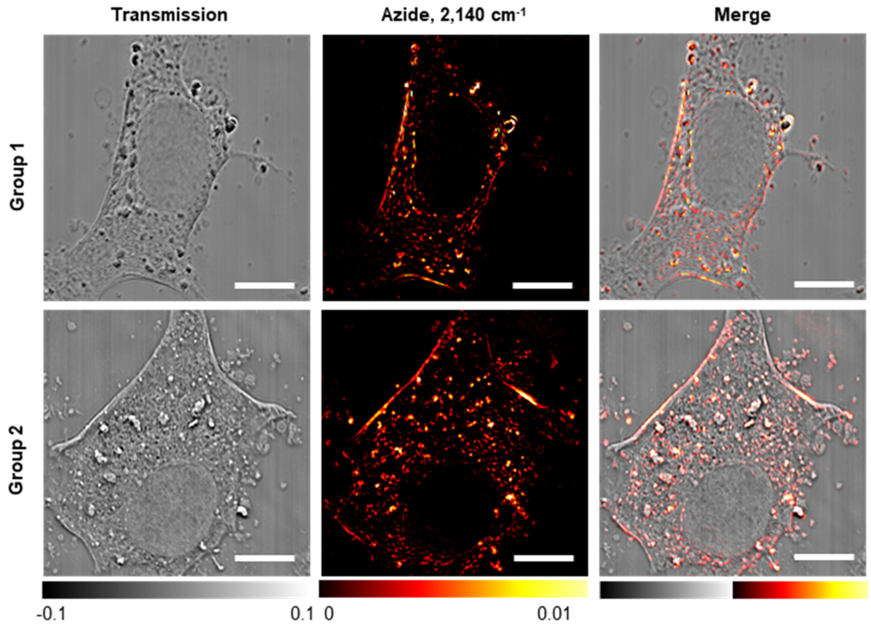

Fig. S17. MIP images of HeLa cells treated with $Ac_4ManNAz$. The azide-conjugated carbohydrates on the cell surface and in the cytoplasm were clearly seen. Scale bars: 20 µm.

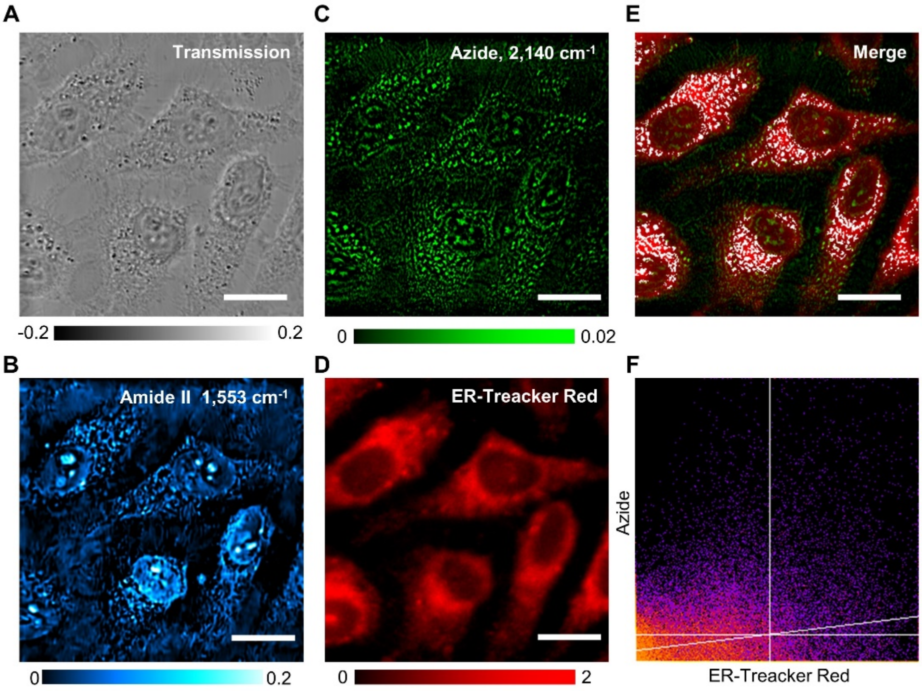

Fig. S18. Colocalized MIP and fluorescence imaging of HeLa cells treated with 50 µM Ac$_4$ManNAz for 40 hours and labeled with ER-Tracker Red dye. (**A**) Transmission and (**B-C**) MIP imaging of HeLa cells in (**B**) protein and (**C**) azide channels. (**D**) Fluorescence imaging of HeLa cells in ER-Tracker Red channel. (**E**) Colocalized MIP and fluorescence imaging of HeLa cells. Scale bar: 20 µm. (**F**) Colocalization analysis of the mapping in (**C**) and (**D**). Pearson's r = 0.829, indicating high spatial correlation.

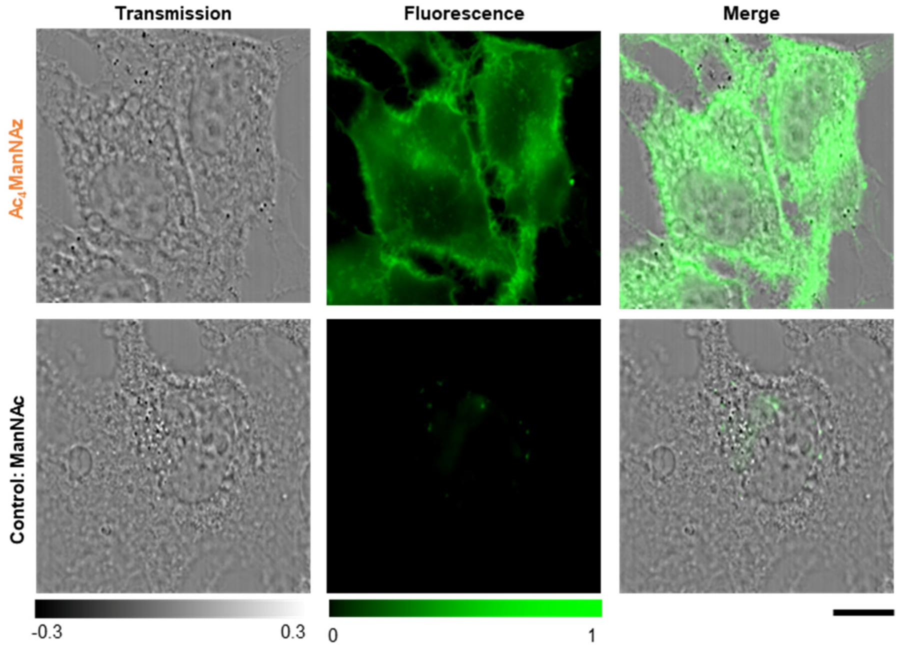

Fig. S19. Fluorescence images of HeLa cells treated with Ac_4ManNAz and ManNAc and underwent click reactions. After 40-hour treatment of Ac_4ManNAz or ManNAc, the cells underwent click reactions with 1-hour incubation of 50 µM AF 488 DBCO. Scale bar: 20 µm.

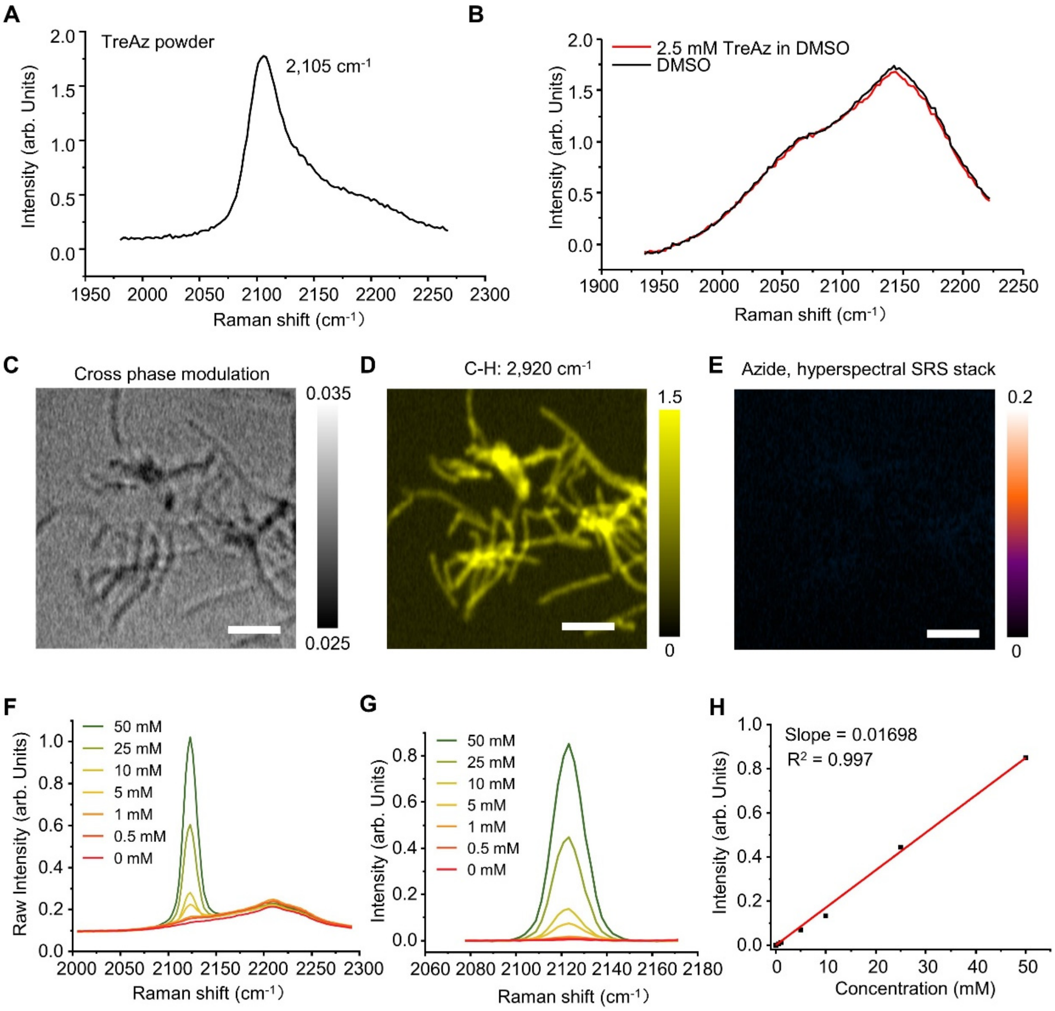

Fig. S20. Stimulated Raman scattering (SRS) spectroscopy and imaging of TreAz. (**A**) SRS spectrum of pure TreAz powder, the azide peak is at 2,105 cm^{-1}. The pump beam wavelength was centered at 851 nm, and the power was 15 mW on sample. The probe beam wavelength was centered at 1,040 nm, and the power was 150 mW on sample. The pixel dwell time was 10 µs. Signals were averaged from 200 by 200 pixels. (**B**) SRS spectra of 2.5 mM TreAz dissolved in DMSO and pure DMSO. The azide peak at the silent window is not detectable. The pump beam wavelength was centered at 851 nm, and the power was 15 mW on sample. The probe beam wavelength was centered at 1,040 nm, and the power was 150 mW on sample. The pixel dwell time was 100 µs. (**C**) Cross phase modulation imaging of *M. smeg* used as a reference. (**D-E**) Hyperspectral SRS imaging at the C-H channel (**D**) and the azide channel (**E**) of *M. smeg* treated with 50 µM TreAz for 26 hours. The C-H channel was recorded at 2,920 cm^{-1}. The Azide channel is a stack sum of 120-frame hyperspectral SRS image from 1,935.2 cm^{-1} to 2,222.3 cm^{-1}. Scale bars: 5 µm. The pump beam wavelength was centered at 851 nm, and the power was 15 mW on sample. The probe beam wavelength was centered at 1,040 nm, and the power was 150 mW on sample. The pixel dwell time was 10 µs. Spectral focusing (*66*) was used to perform hyperspectral SRS imaging. A water objective with magnification of 60 and NA of 1.2 was used for focusing. An oil condenser with NA of 1.4 was used for signal collection. (**F**) SRS signal with gradient

concentrations of EdU dissolved in DMSO, the alkyne peak is at 2,123 cm^{-1}. The pump beam wavelength was centered at 851 nm, and the power was 15 mW on sample. The probe beam wavelength was centered at 1,040 nm, and the power was 150 mW on sample. The pixel dwell time was 10 μs. Signals were averaged from 200 by 200 pixels. (**G**) SRS signal with gradient concentrations of EdU dissolved in DMSO after baseline correction. (**H**) Limit of detection (LOD) of SRS for EdU in DMSO. LOD = 3σ/Slope = 276 μM, where σ is the standard deviation of the baseline for pure DMSO (σ = 0.00156) and Slope is the slope of the intensity-concentration linear calibration curve (Slope = 0.01698).

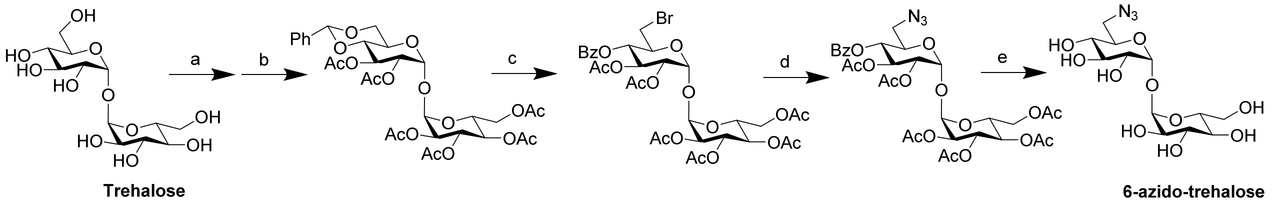

Fig. S21. Synthetic procedures of TreAz. (a) $PhCH(OCH_3)_2$, PTS, DMF, 25 °C for 18 h and 40 °C for 4 h; (b) Et_3N, Ac_2O, DMAP, DMF, 25 °C, overnight (40% overall yield from combined steps of a and b); (c) NBS, $CaCO_3$, trifluorotoluene, 77 °C, 5 hours (80%); (d) NaN_3, DMF, Ar, 65 °C, 5 hours (77%); (e) $NaOCH_3$, CH_3OH, 25 °C, 1.5 hours (97%).

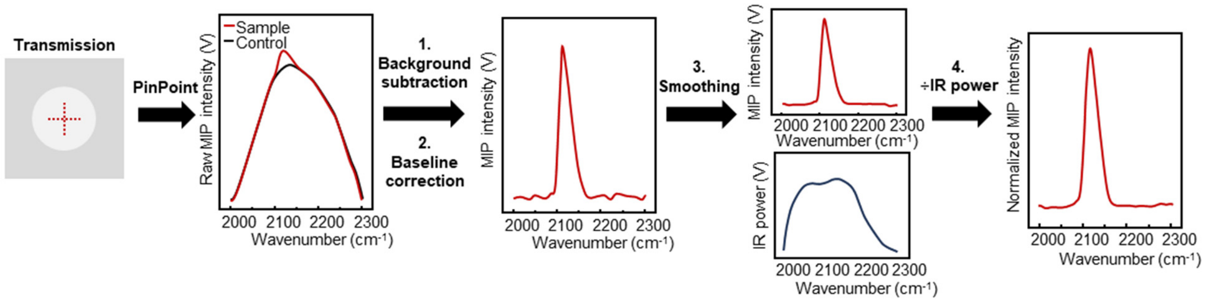

Fig. S22. Pinpoint MIP spectra processing workflow.

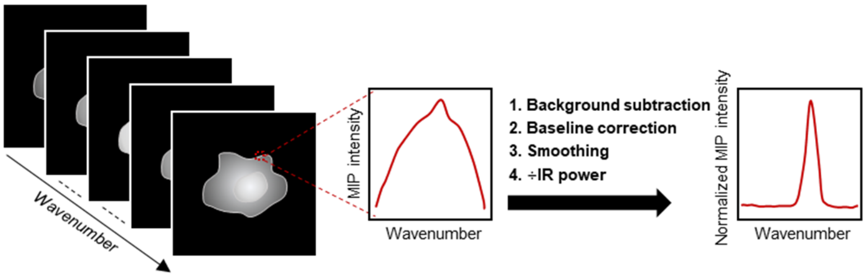

Fig. S23. Hyperspectral MIP spectra processing workflow.

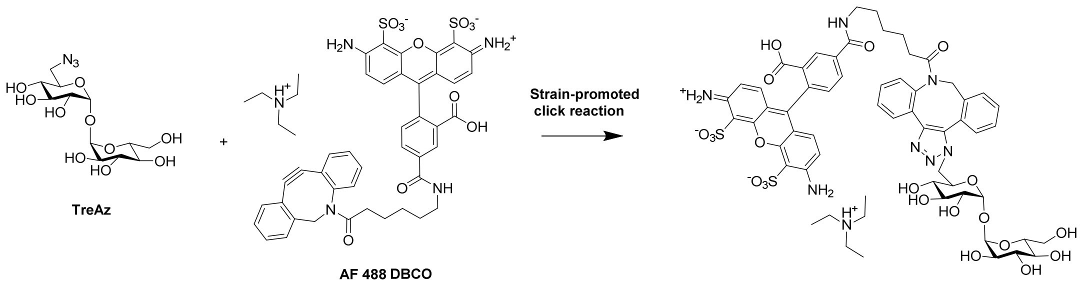

Fig. S24. The scheme of click reaction.

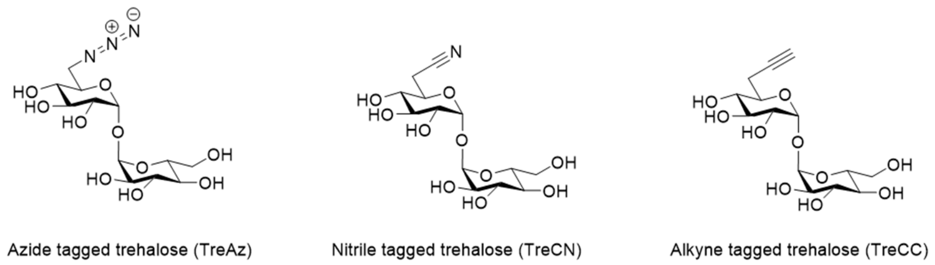

Fig. S25. Molecular structures of trehalose analogs.

Table S1. IR absorption peak cross sections of azide, nitrile and alkyne conjugated to trehalose. Calculations were performed using the B3LYP/6-311G** level of theory (*67-69*) with the Gaussian 16 Revision C.01 Program. Initial structures were built with Avogadro Version 1.2.0 (*70*). These structures were then optimized by Gaussian, prior to a frequency calculation. Molecular structures are shown in **Fig. S25**.

Trehalose analogs	IR Cross section (10^{-18} cm^2/molecule)
TreAz	4.462
TreCN	0.258
TreCC	0.0378

REFERENCES AND NOTES

1. C. Reily, T. J. Stewart, M. B. Renfrow, J. Novak, Glycosylation in health and disease. *Nat. Rev. Nephrol.* **15**, 346–366 (2019).

2. E. Cabib, J. Arroyo, How carbohydrates sculpt cells: Chemical control of morphogenesis in the yeast cell wall. *Nat. Rev. Microbiol.* **11**, 648–655 (2013).

3. B. M. Swarts, C. M. Holsclaw, J. C. Jewett, M. Alber, D. M. Fox, M. S. Siegrist, J. A. Leary, R. Kalscheuer, C. R. Bertozzi, Probing the mycobacterial trehalome with bioorthogonal chemistry. *J. Am. Chem. Soc.* **134**, 16123–16126 (2012).

4. J. Hofmann, H. S. Hahm, P. H. Seeberger, K. Pagel, Identification of carbohydrate anomers using ion mobility–mass spectrometry. *Nature* **526**, 241–244 (2015).

5. A. Barattucci, C. M. A. Gangemi, A. Santoro, S. Campagna, F. Puntoriero, P. Bonaccorsi, Bodipy-carbohydrate systems: Synthesis and bio-applications. *Org. Biomol. Chem.* **20**, 2742–2763 (2022).

6. B. Thomas, K. C. Yan, X. L. Hu, M. Donnier-Marechal, G. R. Chen, X. P. He, S. Vidal, Fluorescent glycoconjugates and their applications. *Chem. Soc. Rev.* **49**, 593–641 (2020).

7. K. E. Hamilton, M. F. Bouwer, L. L. Louters, B. D. Looyenga, Cellular binding and uptake of fluorescent glucose analogs 2-NBDG and 6-NBDG occurs independent of membrane glucose transporters. *Biochimie* **190**, 1–11 (2021).

8. A. Zlitni, G. Gowrishankar, I. Steinberg, T. Haywood, S. Sam Gambhir, Maltotriose-based probes for fluorescence and photoacoustic imaging of bacterial infections. *Nat. Commun.* **11**, 1250 (2020).

9. K. M. Backus, H. I. Boshoff, C. S. Barry, O. Boutureira, M. K. Patel, F. D'Hooge, S. S. Lee, L. E. Via, K. Tahlan, C. E. Barry, B. G. Davis, Uptake of unnatural trehalose analogs as a reporter for *Mycobacterium tuberculosis*. *Nat. Chem. Biol.* **7**, 228–235 (2011).

10. Y. Shen, F. Hu, W. Min, Raman imaging of small biomolecules. *Annu. Rev. Biophys.* **48**, 347–369 (2019).

11. N. Banahene, H. W. Kavunja, B. M. Swarts, Chemical reporters for bacterial glycans: Development and applications. *Chem. Rev.* **122**, 3336–3413 (2022).

12. J. A. Prescher, C. R. Bertozzi, Chemistry in living systems. *Nat. Chem. Biol.* **1**, 13–21 (2005).

13. Kenry, B. Bio-orthogonal click chemistry for In vivo bioimaging. *Trends Chem.* **1**, 763–778 (2019).

14. M. J. Hangauer, C. R. Bertozzi, A FRET-based fluorogenic phosphine for live-cell imaging with the staudinger ligation. *Angew. Chem. Int. Ed. Engl.* **47**, 2394–2397 (2008).

15. J. M. Baskin, J. A. Prescher, S. T. Laughlin, N. J. Agard, P. V. Chang, I. A. Miller, A. Lo, J. A. Codelli, C. R. Bertozzi, Copper-free click chemistry for dynamicin vivoimaging. *Proc. Natl. Acad. Sci. U.S.A.* **104**, 16793–16797 (2007).

16. M. Boyce, I. S. Carrico, A. S. Ganguli, S. H. Yu, M. J. Hangauer, S. C. Hubbard, J. J. Kohler, C. R. Bertozzi, Metabolic cross-talk allows labeling of O-linked β-*N*-acetylglucosamine-modified proteins via the N-acetylgalactosamine salvage pathway. *Proc. Natl. Acad. Sci. U.S.A.* **108**, 3141–3146 (2011).

17. N. Geva-Zatorsky, D. Alvarez, J. E. Hudak, N. C. Reading, D. Erturk-Hasdemir, S. Dasgupta, U. H. von Andrian, D. L. Kasper, In vivo imaging and tracking of host–microbiota interactions via metabolic labeling of gut anaerobic bacteria. *Nat. Med.* **21**, 1091–1100 (2015).

18. H. Liang, K. E. DeMeester, C. W. Hou, M. A. Parent, J. L. Caplan, C. L. Grimes, Metabolic labelling of the carbohydrate core in bacterial peptidoglycan and its applications. *Nat. Commun.* **8**, 15015 (2017).

19. L. Wang, M. Tran, E. D'Este, J. Roberti, B. Koch, L. Xue, K. Johnsson, A general strategy to develop cell permeable and fluorogenic probes for multicolour nanoscopy. *Nat. Chem.* **12**, 165–172 (2020).

20. D. Zhang, C. Li, C. Zhang, M. N. Slipchenko, G. Eakins, J. X. Cheng, Depth-resolved mid-infrared photothermal imaging of living cells and organisms with submicrometer spatial resolution. *Sci. Adv.* **2**, e1600521 (2016).

21. Q. Xia, J. Yin, Z. Guo, J. X. Cheng, Mid-Infrared photothermal microscopy: Principle, instrumentation, and applications. *J. Phys. Chem. B* **126**, 8597–8613 (2022).

22. Y. Bai, C. M. Camargo, S. M. K. Glasauer, R. Gifford, X. Tian, A. P. Longhini, K. S. Kosik, Single-cell mapping of lipid metabolites using an infrared probe in human-derived model systems. *Nat. Commun.* **15**, 350 (2024).

23. J. Yin, M. Zhang, Y. Tan, Z. Guo, H. He, L. Lan, J. X. Cheng, Video-rate mid-infrared photothermal imaging by single-pulse photothermal detection per pixel. *Sci. Adv.* **9**, eadg8814 (2023).

24. S. A. Wijesundera, S. H. Liyanage, P. Biswas, J. F. Reuther, M. Yan, Trehalose-grafted glycopolymer: Synthesis via the staudinger reaction and capture of Mycobacteria. *Biomacromolecules* **24**, 238–245 (2023).

25. X. S. Gai, B. A. Coutifaris, S. H. Brewer, E. E. Fenlon, A direct comparison of azide and nitrile vibrational probes. *Phys. Chem. Chem. Phys.* **13**, 5926–5930 (2011).

26. K. I. Oh, J. H. Lee, C. Joo, H. Han, M. Cho, β-Azidoalanine as an IR probe: Application to amyloid Aβ(16-22) aggregation. *J. Phys. Chem. B* **112**, 10352–10357 (2008).

27. S. M. Salehi, D. Koner, M. Meuwly, Vibrational spectroscopy of N_3^- in the gas and condensed phase. *J. Phys. Chem. B* **123**, 3282–3290 (2019).

28. M. Lim, P. Hamm, R. M. Hochstrasser, Protein fluctuations are sensed by stimulated infrared echoes of the vibrations of carbon monoxide and azide probes. *Proc. Natl. Acad. Sci. U.S.A.* **95**, 15315–15320 (1998).

29. M. Garcia-Viloca, K. Nam, C. Alhambra, J. L. Gao, Solvent and protein effects on the vibrational frequency shift and energy relaxation of the azide ligand in carbonic anhydrase. *J. Phys. Chem. B* **108**, 13501–13512 (2004).

30. C. Lima, H. Muhamadali, Y. Xu, M. Kansiz, R. Goodacre, Imaging isotopically labeled bacteria at the single-cell level using high-resolution optical infrared photothermal spectroscopy. *Anal. Chem.* **93**, 3082–3088 (2021).

31. F. Tai, K. Koike, H. Kawagoe, J. Ando, Y. Kumamoto, N. I. Smith, M. Sodeoka, K. Fujita, Detecting nitrile-containing small molecules by infrared photothermal microscopy. *Analyst* **146**, 2307–2312 (2021).

32. H. Ni, Y. Yuan, M. Li, Y. Zhu, X. Ge, C. V. P. Dessai, L. Wang, J. X. Cheng, Millimeter-deep micron-resolution vibrational imaging by shortwave infrared photothermal microscopy. arXiv:2310.05798 (2023).

33. M. Jackson, C. M. Stevens, L. Zhang, H. I. Zgurskaya, M. Niederweis, Transporters involved in the biogenesis and functionalization of the mycobacterial cell envelope. *Chem. Rev.* **121**, 5124–5157 (2021).

34. R. L. Nieto, C. Mehaffy, M. N. Islam, B. Fitzgerald, J. Belisle, J. Prenni, K. Dobos, Biochemical characterization of isoniazid-resistant *Mycobacterium tuberculosis*: Can the analysis of clonal strains reveal novel targetable pathways? *Mol. Cell. Proteomics* **17**, 1685–1701 (2018).

35. T. Plass, S. Milles, C. Koehler, C. Schultz, E. A. Lemke, Genetically encoded copper-free click chemistry. *Angew. Chem. Int. Ed. Engl.* **50**, 3878–3881 (2011).

36. Craig S. McKay, M. G. Click chemistry in complex mixtures: Bioorthogonal bioconjugation. *Chem. Biol.* **21**, 1075–1101 (2014).

37. Z. J. Witczak, in *Carbohydrate Chemistry*, A. Pilar Rauter, T. Lindhorst, Eds. (The Royal Society of Chemistry, 2010), vol. 36, pp. 176–193.

38. S. Cecioni, D. Goyard, J.-P. Praly, S. Vidal, in *Carbohydrate Microarrays: Methods and Protocols*, Y. Chevolot, Ed. (Humana Press, 2012), pp. 57–68.

39. R. Caputto, L. F. Leloir, R. E. Trucco, C. E. Cardini, A. C. Paladini, The enzymatic transformation of galactose into glucose derivatives. *J. Biol. Chem.* **179**, 497–498 (1949).

40. C. A. Sellick, R. N. Campbell, R. J. Reece, in *International Review of Cell and Molecular Biology* (Academic Press, 2008), vol. 269, pp. 111–150.

41. J. B. Thoden, H. M. Holden, The molecular architecture of galactose mutarotase/UDP-galactose 4-epimerase from *Saccharomyces cerevisiae*. *J. Biol. Chem.* **280**, 21900–21907 (2005).

42. M. Kufleitner, L. M. Haiber, V. Wittmann, Metabolic glycoengineering-exploring glycosylation with bioorthogonal chemistry. *Chem. Soc. Rev.* **52**, 510–535 (2023).

43. P. V. Chang, J. A. Prescher, E. M. Sletten, J. M. Baskin, I. A. Miller, N. J. Agard, A. Lo, C. R. Bertozzi, Copper-free click chemistry in living animals. *Proc. Natl. Acad. Sci. U.S.A.* **107**, 1821–1826 (2010).

44. C. R. Bertozzi, L. L. Kiessling, Chemical glycobiology. *Science* **291**, 2357–2364 (2001).

45. H. Yamakoshi, K. Dodo, M. Okada, J. Ando, A. Palonpon, K. Fujita, S. Kawata, M. Sodeoka, Imaging of EdU, an alkyne-tagged cell proliferation probe, by Raman microscopy. *J. Am. Chem. Soc.* **133**, 6102–6105 (2011).

46. H. Yamakoshi, K. Dodo, A. Palonpon, J. Ando, K. Fujita, S. Kawata, M. Sodeoka, Alkyne-tag Raman imaging for visualization of mobile small molecules in live cells. *J. Am. Chem. Soc.* **134**, 20681–20689 (2012).

47. L. Wei, F. Hu, Y. Shen, Z. Chen, Y. Yu, C. C. Lin, M. C. Wang, W. Min, Live-cell imaging of alkyne-tagged small biomolecules by stimulated Raman scattering. *Nat. Methods* **11**, 410–412 (2014).

48. S. Hong, T. Chen, Y. Zhu, A. Li, Y. Huang, X. Chen, Live-cell stimulated Raman scattering imaging of alkyne-tagged biomolecules. *Angew. Chem. Int. Ed.* **53**, 5827–5831 (2014).

49. F. Hu, L. Shi, W. Min, Biological imaging of chemical bonds by stimulated Raman scattering microscopy. *Nat. Methods* **16**, 830–842 (2019).

50. H. Fujioka, J. Shou, R. Kojima, Y. Urano, Y. Ozeki, M. Kamiya, Multicolor activatable Raman probes for simultaneous detection of plural enzyme activities. *J. Am. Chem. Soc.* **142**, 20701–20707 (2020).

51. J. Ao, X. Fang, X. Miao, J. Ling, H. Kang, S. Park, C. Wu, M. Ji, Switchable stimulated Raman scattering microscopy with photochromic vibrational probes. *Nat. Commun.* **12**, 3089 (2021).

52. J. Shou, A. Komazawa, Y. Wachi, M. Kawatani, H. Fujioka, S. J. Spratt, T. Mizuguchi, K. Oguchi, H. Akaboshi, F. Obata, R. Tachibana, S. Yasunaga, Y. Mita, Y. Misawa, R. Kojima, Y. Urano, M. Kamiya, Y. Ozeki, Super-resolution vibrational imaging based on photoswitchable Raman probe. *Sci. Adv.* **9**, eade9118 (2023).

53. J. Ma, I. M. Pazos, W. Zhang, R. M. Culik, F. Gai, Site-specific infrared probes of proteins. *Annu. Rev. Phys. Chem.* **66**, 357–377 (2015).

54. L. Shi, X. Liu, L. Shi, H. T. Stinson, J. Rowlette, L. J. Kahl, C. R. Evans, C. Zheng, L. E. P. Dietrich, W. Min, Mid-infrared metabolic imaging with vibrational probes. *Nat. Methods* **17**, 844–851 (2020).

55. X. Liu, L. Shi, Z. Zhao, J. Shu, W. Min, VIBRANT: Spectral profiling for single-cell drug responses. *Nat. Methods* **21**, 501–511 (2024).

56. X. Gao, X. Li, W. Min, Absolute stimulated Raman cross sections of molecules. *J. Phys. Chem. Lett.* **14**, 5701–5708 (2023).

57. S. Ramos, J. C. Lee, Water bend-libration as a cellular Raman imaging probe of hydration. *Proc. Natl. Acad. Sci. U.S.A.* **120**, e2313133120 (2023).

58. J. Yin, L. Lan, Y. Zhang, H. Ni, Y. Tan, M. Zhang, Y. Bai, J. X. Cheng, Nanosecond-resolution photothermal dynamic imaging via MHZ digitization and match filtering. *Nat. Commun.* **12**, 7097 (2021).

59. P. D. Samolis, X. Zhu, M. Y. Sander, Time-resolved mid-infrared photothermal microscopy for imaging water-embedded axon bundles. *Anal. Chem.* **95**, 16514–16521 (2023).

60. L. Z. Osherovich, B. S. Cox, M. F. Tuite, J. S. Weissman, Dissection and design of yeast prions. *PLoS Biol.* **2**, E86 (2004).

61. S. H. Liyanage, N. G. H. Raviranga, J. G. Ryan, S. S. Shell, O. Ramstrom, R. Kalscheuer, M. Yan, Azide-masked fluorescence turn-on probe for imaging mycobacteria. *JACS Au* **3**, 1017–1028 (2023).

62. H. He, J. Yin, M. Li, C. V. P. Dessai, M. Yi, X. Teng, M. Zhang, Y. Li, Z. Du, B. Xu, J. X. Cheng, Mapping enzyme activity in living systems by real-time mid-infrared photothermal imaging of nitrile chameleons. *Nat. Methods* **21**, 342–352 (2024).

63. K. Dabov, A. Foi, V. Katkovnik, K. Egiazarian, Image denoising by sparse 3-D transform-domain collaborative filtering. *IEEE Trans. Image Process.* **16**, 2080–2095 (2007).

64. M. Maggioni, G. Boracchi, A. Foi, K. Egiazarian, Video denoising, deblocking, and enhancement through separable 4-D nonlocal spatiotemporal transforms. *IEEE Trans. Image Process.* **21**, 3952–3966 (2012).

65. E. Kim, H. Koo, Biomedical applications of copper-free click chemistry: In vitro, in vivo, and ex vivo. *Chem. Sci.* **10**, 7835–7851 (2019).

66. D. Fu, G. Holtom, C. Freudiger, X. Zhang, X. S. Xie, Hyperspectral imaging with stimulated Raman scattering by chirped femtosecond lasers. *J. Phys. Chem. B* **117**, 4634–4640 (2013).

67. A. D. Becke, Density-functional thermochemistry. III. The role of exact exchange. *J. Chem. Phys.* **98**, 5648–5652 (1993).

68. C. Lee, W. Yang, R. G. Parr, Development of the Colle-Salvetti correlation-energy formula into a functional of the electron density. *Phys. Rev. B Condens Matter* **37**, 785–789 (1988).

69. R. Krishnan, J. S. Binkley, R. Seeger, J. A. Pople, Self-consistent molecular orbital methods. XX. A basis set for correlated wave functions. *J. Chem. Phys.* **72**, 650–654 (1980).

70. M. D. Hanwell, D. E. Curtis, D. C. Lonie, T. Vandermeersch, E. Zurek, G. R. Hutchison, Avogadro: An advanced semantic chemical editor, visualization, and analysis platform. *J. Cheminform.* **4**, 17 (2012).
